# Supplementary material for: Chromothripsis during telomere crisis is independent of NHEJ, and consistent with a replicative origin
Source: Genome Res. 2019 May;29(5):737–49. doi: 10.1101/gr.240705.118 (PMC6499312; doi:10.1101/gr.240705.118)
Supplement: Supplemental Material [file supp_gr.240705.118_Supplemental_file_1.zip › contigs/annotated_contigs/DB86/contig.2.DB86_length_507_mean_cov_5.51084812623.docx]

**DB86_length_507_mean_cov_5.51084812623**

```
TGATATATAAGGCATCTGTGAAATGAGATGCTGGAAGAAATACATATATAAATGAATAAATTGAGAGCAAATAAGGGCCAAATTTCAGA
 >chr3:82109510-82109772 - E=4e-146 p=2e-03
AAGACTAAACTCTTGTAAACTGAATATGAAGGCCTACAATGCTTATCAAGTCAGAAAAAATGTAATCTATTCAAAATGGAATCATAGAT

TTACTTGGGTTCAGCTTTATGGCCTCCTTCAATCCAGGTTGACATTATTTGTGCAACACTGACTTAAAAAGTGT|TTTTAGTACA|GTA
                                                                           >chr16:80912
TAGAGTAAAAATAGATTATAGTAAAAAGTATTTTTTTAATTTAAATTGTTTGTGTATTGGCCAGGCACAGTGGCTCACACCCATAATCT
658-80912913 + E=7e-139
CAGCAATTTGGGAGGCCAAGACGAGTGGATCATTTGAGATGAGGAGTTTGAGACCAGCCTGACCAACATGGTGAAACCCCATCTCTACT

AAAAATGCAAAAAAAAATTAGCCAGGCATGGTGGTGCATGCCTGTAATCCCAGCTACTTGGGAG
```
